# Marriage intentions, desires, and pathways to later and less marriage in Japan

**DOI:** 10.4054/demres.2021.44.3

**Published:** 2021-01-12

**Authors:** James M. Raymo, Fumiya Uchikoshi, Shohei Yoda

**Affiliations:** 1 Department of Sociology and Office of Population Research, Princeton University.; 2 Department of Sociology and Office of Population Research, Princeton University.; 3 National Institute of Population and Social Security Research, Tokyo.

## Abstract

**BACKGROUND:**

Understanding the trend toward later and less marriage is particularly important in low-fertility societies where alternatives to marriage are limited and childbearing outside of marriage remains rare.

**OBJECTIVE:**

Our goal in this paper is to advance our understanding of the wide variety of explanations offered for later and less marriage in Japan by focusing explicitly on marriage intentions and desires.

**METHODS:**

Using two sources of nationally representative data, we describe the prevalence of positive, negative, and passive marriage intentions and desires among men and women who have never been married. We also examine socioeconomic differences in intentions, patterns of marriage desires across young adulthood, and relationships between marriage desires and outcomes. By linking three pathways to later and less marriage (rejection of marriage, failure to realize marriage desires, and unplanned drifting into singlehood) to specific theoretical frameworks, we generate indirect insights into explanations for later and less marriage.

**CONCLUSIONS:**

Although the large majority of unmarried men and women want to marry, less than half of respondents married across nine waves of the Japanese Life Course Panel Survey. Among those who remained unmarried, roughly two-thirds can be classified as ‘drifting’ into singlehood, about 30% as ‘failing to realize marriage desires,’ and no more than 5% as ‘rejecting marriage.’

**CONTRIBUTION:**

By extending the small body of research on marriage intentions and desires, this study provides a framework for thinking broadly about explanations for later and less marriage in Japan and highlights the importance of both failure to realize marriage desires and unplanned drifting into singlehood.

## 1. Introduction

The trend toward later and less marriage in East Asian societies is dramatic. In Japan, where marriage was, until recently, universal and concentrated in a narrow band of ‘appropriate’ ages (Brinton 1992), the mean age of first marriage is now 31 for men and 29 for women, and 20% of women are projected to never marry (National Institute of Population and Social Security Research, IPSS hereafter, 2017a; 2019). These trends are not unique to Japan (or East Asia) but are arguably more consequential in rapidly aging, low-fertility societies where the relationship between marriage and childbearing remains strong. Later and less marriage is inextricably linked to Japan’s low fertility rates given that marital fertility has remained stable (at around two children) and levels of nonmarital childbearing remain negligibly low (e.g., Tsuya and Mason 1995). Indeed, scholars have stressed the importance of understanding marriage and childbearing as jointly determined components of the family formation process in Japan (Rindfuss et al. 2004), with the desire to have children (or pregnancy itself) a primary motivation for marriage and marriage almost invariably involving parenthood.

Explanations for declining marriage rates in Japan include, but are certainly not limited to, attitudinal change, the high costs of children, the comforts of single life, and the difficulties of balancing work and family in a highly gender-inegalitarian society. A comprehensive evaluation of the varied and complex array of explanations examined in past research is beyond the scope of any single study, but our goal in this paper is to take a step in this direction. We begin by recognizing that the large body of existing literature can be effectively and succinctly summarized using a small group of theoretically informed pathways to later and less marriage.^4^ Here, we use the term pathways to represent patterns of experience across early adulthood and middle age that are associated with delayed marriage and lifelong singlehood, either intentionally or unintentionally.

This approach not only allows us to effectively summarize diverse theoretical predictions but also motivates our focus on marriage intentions and desires.^5^ We consider the marriage intentions and desires of both women and men; describe the prevalence of positive, negative, and passive intentions/desires; and examine socioeconomic differences in intentions/desires, patterns of marriage desires across young adulthood, and relationships between marriage desires and outcomes. It is our linking of the three pathways to later and less marriage – rejection of marriage, failure to realize marriage desires, and unplanned drifting into singlehood – to specific predictions about socioeconomic differences in marriage intentions/desires, patterns across young adulthood, and marriage outcomes that allows for evaluation of insights from a range of theoretical perspectives.

## 2. Background: Pathways to later and less marriage

### 2.1 Rejection of marriage

Earlier studies of marriage in Japan consistently demonstrated that women’s educational attainment and earnings were negatively associated with the likelihood of ever marrying (Ono 2003; Raymo 1998, 2003; Retherford, Ogawa, and Matsukura 2001; Tsuya 2006). These results suggest a retreat from, or rejection of, marriage among high socioeconomic status (SES) women, a scenario that would be consistent with two prominent theoretical frameworks. The first, and perhaps most commonly referenced, framework for understanding declining rates of marriage in developed countries argues that growing economic independence for women reduces the gains to marriage that can be derived from spouses’ pooling of complementary specializations (Becker 1981). In a series of influential papers, Oppenheimer (1988, 1994, 1997) compellingly argues that this ‘specialization and trading’ model is logically a theory of nonmarriage that implies women will use their increased economic independence to ‘buy out’ of marriages that entail substantial opportunity costs. To the extent that buying out of marriage is an active, intentional choice, this theoretical framework suggests that intentions and desires to remain unmarried should be most prevalent among women for whom the opportunity costs of marriage are greatest (i.e., those with college education, high earnings, and professional occupations).^6^ It also suggests that intentions/desires to remain unmarried should be stable across young adulthood and associated with a low risk of ever marrying.

A second theoretical framework consistent with rejection of marriage is that of the second demographic transition. This influential framework describes a shift in values and life orientations in which marriage becomes a purposeful individual choice rather than an unquestioned, or even obligatory, part of life (Lesthaeghe 1995, 2010; Surkyn and Lesthaeghe 2004). In contrast to the independence hypothesis, which provides little insight regarding men’s marriage intentions/desires, writing on the second demographic transition suggests that rejection of marriage should be particularly salient among both men and women belonging to a group of highly educated innovators.^7^ To the extent that individuation, secularization, increasing gender egalitarianism, and rising consumption aspirations central to this framework reduce the attractiveness of marriage as a life choice, we should expect to see relatively high and stable intentions/desires to remain unmarried and lower rates of marriage among men and women of higher socioeconomic status. However, there are reasons to question how well Japan’s experience corresponds to conventional depictions of the second demographic transition (e.g., Atoh, Kandiah, and Ivanov 2004; Matsuo 2001). These include relatively limited change in life orientation or individuation (Atoh 2001) and the fact that lifelong singlehood for men is concentrated among those with lower levels of education (Fukuda, Raymo, and Yoda 2020). Nevertheless, we see value in considering the relevance of this widely cited framework.

### 2.2 Failure to realize intentions/desires to marry

While the economic independence hypothesis and second demographic transition framework provide explanations for later and less marriage that appear consistent with findings from previous research on Japan, attitudinal data offer little evidence of a widespread rejection of marriage. Recent data show that 86% of men who have never married and 89% of women who have never married say that they intend to marry at some point (IPSS 2017b). Other studies of marriage desires also indicate that a large majority of Japanese women (and men) who have never married wants to marry at some point (Inaba et al. 2015; Miwa 2019; Mizuochi, Tsutsui, and Asai 2010) and that stronger desire to marry is positively associated with entry into both nonmarital partnerships and marriage (Motegi and Ishida 2019).

The theory of planned behavior (e.g., Ajzen 1985, 1991) suggests that the pronounced gap in Japan between near-universal intentions to marry and growing proportions who never marry reflects external factors that limit individual control over marriage behavior. Consistent with this theoretical expectation, previous research has emphasized a range of social and economic factors that may act as barriers to realization of the intentions to marry articulated by unmarried Japanese men and women. These include a rise in precarious nonstandard employment for both women and men (Piotrowski, Kalleberg, and Rindfuss 2015; Raymo and Shibata 2017), mismatches in the marriage market detrimental to highly educated women and low-educated men (Fukuda, Raymo, and Yoda 2020; Miwa 2019; Raymo and Iwasawa 2005), and the combination of decline in facilitated (*miai*) marriages, sex-segregation in the workplace, and the waning salience of the workplace as a venue for meeting potential partners (Iwasawa and Mita 2007).

The complex array of life circumstances through which young adults who want to marry end up ‘drifting’ into lifelong singlehood is compellingly articulated in a recent study of women in Tokyo (Yoshida 2017). The life histories of Yoshida’s (2017) respondents can be summarized as two distinct types of drifting into singlehood. In one, women’s intentions or desires to marry are derailed by the precarious life circumstances of their potential partners and/or by their own economic difficulties, family obligations, or bad luck. In the other, women approach marriage passively in young adulthood, focusing their time and energy on work and other pursuits, vaguely assuming that they will marry in their late twenties or early thirties, but investing relatively little in romantic relationships. We refer to the first scenario as ‘failing to realize marriage intentions/desires’ and the second as ‘unplanned drifting into singlehood.’

Research on the gap between marriage desires and outcomes in the United States has placed primary emphasis on low wages and economic insecurity at young ages (Smock, Manning, and Porter 2005). This work references the difficulty of entering stable employment (Oppenheimer, Kalmijn, and Lim 1997), increases in involuntary job loss (Farber 2005), declining labor union membership (Schneider and Reich 2014), and a rise in ‘bad jobs’ characterized by low pay, limited employment security, and no benefits (Kalleberg 2009). The impact of these changes is thought to be particularly salient for men and women with lower levels of education who, when unable to reach the perceived economic ‘bar’ for marriage (Gibson-Davis, Edin, and McLanahan 2005) or to achieve the stability required for marriage as a ‘capstone’ event (Cherlin 2004), increasingly enter cohabiting unions (Lundberg, Pollak, and Stearns 2016; Smock, Manning, and Porter 2005) and have children outside of marriage (Perelli-Harris and Lyons-Amos 2016). Similar changes in Japan, where these nonmarital family alternatives are limited, are arguably more likely to lead to an increase in lifelong singlehood.

The shifting employment landscape may also impact marriage via the marriage market and the spouse search process, with less-educated men finding it difficult to attract potential spouses. In the United States, this focus on the shrinking pool of ‘marriageable men’ has featured prominently in research on declining rates of marriage among Black women (e.g., Lichter et al. 1992), and a similar argument has been made for men with lower levels of education and income in Japan (Fukuda, Raymo, and Yoda 2020; Miwa 2019) and South Korea (Raymo and Park 2020). Increasing uncertainty in men’s economic prospects may have a particularly strong impact on marriage in societies where expectations of a clear gender division of labor within marriage remain strong. Men facing the greatest uncertainty will marry later (and perhaps less) not only because women find them unappealing as potential mates, but also because men themselves perceive the economic responsibilities associated with the role of primary breadwinner to be unattractive or unachievable. Indeed, several of Yoshida’s (2017) respondents attributed failure to realize their own plans for marriage to their male partners’ unstable life circumstances. We thus expect that some men and women, particularly those at the lower end of the socioeconomic distribution, will remain unmarried despite clear and stable intentions to marry.

### 2.3 Unplanned drifting into singlehood

Yoshida’s (2017) depiction of a passive drift into singlehood is potentially consistent with several lines of research on marriage trends in Japan. One prominent example is research emphasizing premarital living arrangements and the comforts of extended residence in the parental home (Raymo and Ono 2007; Yu and Kuo 2016). Related work discusses the growing comforts of single life in urban Japan (Yamada 1999) and perceptions, especially among women, that marriage would necessitate changes in their professional and personal lives that they are not yet ready to make (Brinton and Oh 2019). Unlike the pathways characterized by rejection of marriage and failure to realize marriage intentions/desires, ‘drifting’ into lifelong singlehood suggests the importance of ambivalent or passive attitudes toward marriage (see Mizuochi, Tsutsui, and Asai (2010) for evidence of this pattern in Japan). Indeed, several of the women in Yoshida’s (2017) study vaguely assumed that they would marry someday but postponed thinking seriously about marriage while prioritizing work and other pursuits until they found themselves at an age, and in life circumstances, where marriage appeared less desirable or feasible. We suspect that this pattern of drifting into singlehood has become more common as the salience of normative pressures to marry by a specific age and perceptions of marriage as necessary or as a social obligation decline (Ministry of Health, Labour and Welfare 2012).

Drifting into lifelong singlehood is potentially consistent with at least two widely referenced theoretical frameworks. According to search theories of marriage timing (Oppenheimer 1988) and related discussion of the shifting economic foundations of marriage (Sweeney 2002), a host of social and economic changes have combined to necessitate longer searches in the marriage market. Of particular importance are growing symmetry in women’s and men’s educational and economic opportunities, increasing strategic value of dual-earner marriages, and growing early-career employment instability that make it both more important and more difficult to evaluate the longer-term economic prospects of potential partners (as well as one’s own prospects) at young ages. In this context, strong incentives for engaging in an extended search for the ‘right’ partner can lead men and women to postpone serious consideration of marriage in young adulthood. Low interest in (or ambivalence toward) marriage at young ages could plausibly result in the pattern of drifting described by Yoshida (2017), with women (and men) finding themselves well into their thirties without a partner or prospects for meeting one.

This passive pathway into lifelong singlehood is also potentially consistent with gender equity theory (McDonald 2000a, 2000b, 2009), a compelling framework for understanding later and less marriage in gender-inegalitarian societies, such as Japan. In this framework, tensions between women’s growing employment opportunities and patriarchal family norms, expectations, and obligations result in an either-or choice between career and family. Although both may be desired, later and less marriage can be understood as the result of women increasingly choosing the former over the latter, at least at younger ages. In this scenario, ambivalent or passive marriage intentions should be particularly pronounced for women with the greatest opportunities in the public sphere (e.g., the labor market) and thus the highest opportunity costs of family formation (i.e., the highly educated, high earners, those in more rewarding occupations; see Fuwa and Yagishita (2016) for evidence of this pattern in Japan). A second important insight from this framework is that high-SES women holding passive or ambivalent views of marriage may be less likely to marry than lower-SES women with similar views.

While Yoshida’s (2017) study focuses on women and emphasizes gender inequality associated with patriarchal gender ideology, a similar argument could be made for an analogous male drift into singlehood. The life course implications of marriage are arguably greater for women in Japan (e.g., Yamada 1996), but there is no reason to believe that work demands, the difficulty of meeting potential partners, and the freedom of singlehood are any less relevant for men’s navigation of young adulthood and the family formation process. Indeed, recent data describe a relatively passive approach to marriage among both men and women, with many Japanese who have never married expressing a desire to marry by a certain age, but relatively few reporting that they are currently in any kind of dating relationship. In 2015, the percentages of unmarried men and women who said that they did not have a partner were 70% and 59%, respectively. Among these unpartnered singles, half of both men and women reported that they were uninterested in dating (IPSS 2017b). Despite popular depictions of young adults’ active pursuit of marriage partners (Yamada and Shirakawa 2008), including the proliferation of online dating services (Dalton and Dales 2016; Miwa and Tanaka 2020), nearly half of unmarried singles are not actively seeking a partner (Miwa 2010; Motegi and Ishida 2019), and this appears to be particularly true for men in more precarious economic circumstances (Inaba et al. 2015; Motegi and Ishida 2019).

### 2.4 Hypotheses

The three scenarios just described can be summarized in the form of specific hypotheses as follows in Table 1:

It is important to reiterate that these three patterns are by no means mutually exclusive. The breadth of previous research suggests that there may be multiple pathways to later and less marriage in Japan and one important contribution of our study is to consider the possibility of a ‘perfect storm’ of heterogenous pathways combining to produce rapid increases in lifelong singlehood in gender-inegalitarian and increasingly precarious societies in which marriage and childbearing remain tightly linked.

## 3. Data and method

To evaluate the hypothesized patterns and relationships summarized in Table 1, we use two different sources of data: the Japanese National Fertility Survey (JNFS) and the Japanese Life Course Panel Survey (JLPS).

### 3.1 Japanese National Fertility Survey

To provide an initial descriptive overview of levels and socioeconomic differentials in marriage intentions (columns 1 and 2 in Table 1), we used pooled data from the 8^th^-15^th^ JNFS. These surveys were conducted by the National Institute for Population and Social Security Research in 1982, 1987, 1992, 1997, 2002, 2005, 2010, and 2015. The JNFS is comprised of two separate nationally representative samples: one of married women age 18 to 49 and one of unmarried men and women age 18 to 34 in the 1982 and 1987 surveys and age 18 to 49 beginning in the 1992 survey. We used the data from the surveys of unmarried men and women, each of which asked 2,000 to 5,500 respondents to report their marriage intentions. The primary strength of the JNFS is its information on marriage intentions over a three-decade period.

We focused on a single survey item that asked respondents to provide a dichotomous yes-no response to the following question: “Thinking about your life as a whole, which of the following describes your thoughts on marriage?” The response options are “I intend to marry at some point” and “I have no intention of ever marrying.” Those who did not provide a response are put in a separate category labeled ‘missing.’ To describe socioeconomic differentials in this simple measure of marriage intentions (column 2, Table 1), we tabulated the responses of men and women who have never been married by educational attainment and by employment status. These two measures of socioeconomic status combine to proxy the various factors central to the theoretical frameworks described above (e.g., economic potential, opportunity costs of marriage, ability to fulfill breadwinner role, gender attitudes, and life orientation). Educational attainment is a three-category measure: (1) high school or less, (2) a two-year college or vocational school, and (3) university or more. Employment status is a four-category measure: (1) regular/full time, (2) nonstandard/part-time, (3) self-employed, and (4) not employed.^8^

### 3.2 Japanese Life Course Panel Survey

We then used data from JLPS to evaluate the posited patterns and relationships summarized in the four columns of Table 1. JLPS is a nationally representative longitudinal survey of men and women aged 20 to 40 conducted annually by the Institute of Social Science at the University of Tokyo. Data are currently available for wave 1 (2007) through wave 9 (2015), with detailed information on sampling procedure and data structure provided by Ishida (2013). We began by restricting the sample to men and women who had never married when first interviewed in 2007. In each wave, unmarried respondents were asked the following question about marriage: “What are your thoughts regarding marriage?” Response options were: “I definitely want to marry,” “I want to marry if possible,” “I don’t care if I marry or not,” “I’m not thinking about marriage,” and “I don’t want to marry.” This range of options allows for a more nuanced understanding of how young adults are thinking about marriage relative to the simple yes-no measure in the JNFS and enhances our ability to evaluate the hypothesized relationships summarized in Table 1. We interpreted the first response category as actively positive attitudes toward marriage, the second category as passively positive attitudes, the third and fourth responses as ambivalent attitudes or low interest in marriage, and the fifth category as rejection of marriage. It is important to stress that this is a question about marriage desires, whereas the JNFS question asked about marriage intentions. As noted above, we do not have reason to expect that the relationships summarized in Table 1 will differ for marriage intentions and marriage desires, but we want to remind readers to keep this distinction in mind, understanding that desires and intentions, though related, are not the same thing.

As in analyses of the JNFS data, we began by describing relationships between marriage desires and socioeconomic characteristics. Measures of respondents’ educational attainment and employment status were defined as in the JNFS data, but differ in that they can vary across repeated observations of individual respondents during the nine-year panel period. After describing marriage desires and their correlates (columns 1 and 2 of Table 1), we described the (in)stability of desires across the nine survey waves by tabulating marriage desires in waves *t* and *t*+1 and by constructing river plots to summarize desires over the full nine-year period (column 3 of Table 1). We also used this information to classify respondents at each wave into one of the three pathways summarized in Table 1. Finally, we estimated event history models to examine relationships between marriage desires and the transition to first marriage (column 4, Table 1). These analyses involved using information from the annual surveys to construct person-year records of exposure to the risk of marriage for each individual, which were then used to estimate discrete-time complementary log-log hazard models (Singer and Willett 2003).

Of the 2,365 men and 2,435 women in the original data at wave 1, we omitted those were currently married, widowed, or divorced (n = 2,545). We also omitted cases with missing values for marriage desires, educational attainment, employment status, or year of first marriage (if married). The sample used for the analysis consisted of 1,247 male and 1,091 female respondents who were age 20 to 40 and never married at the time of the first survey in 2007.

## 4. Results

### 4.1 Japanese National Fertility Survey

**Figure 1:**
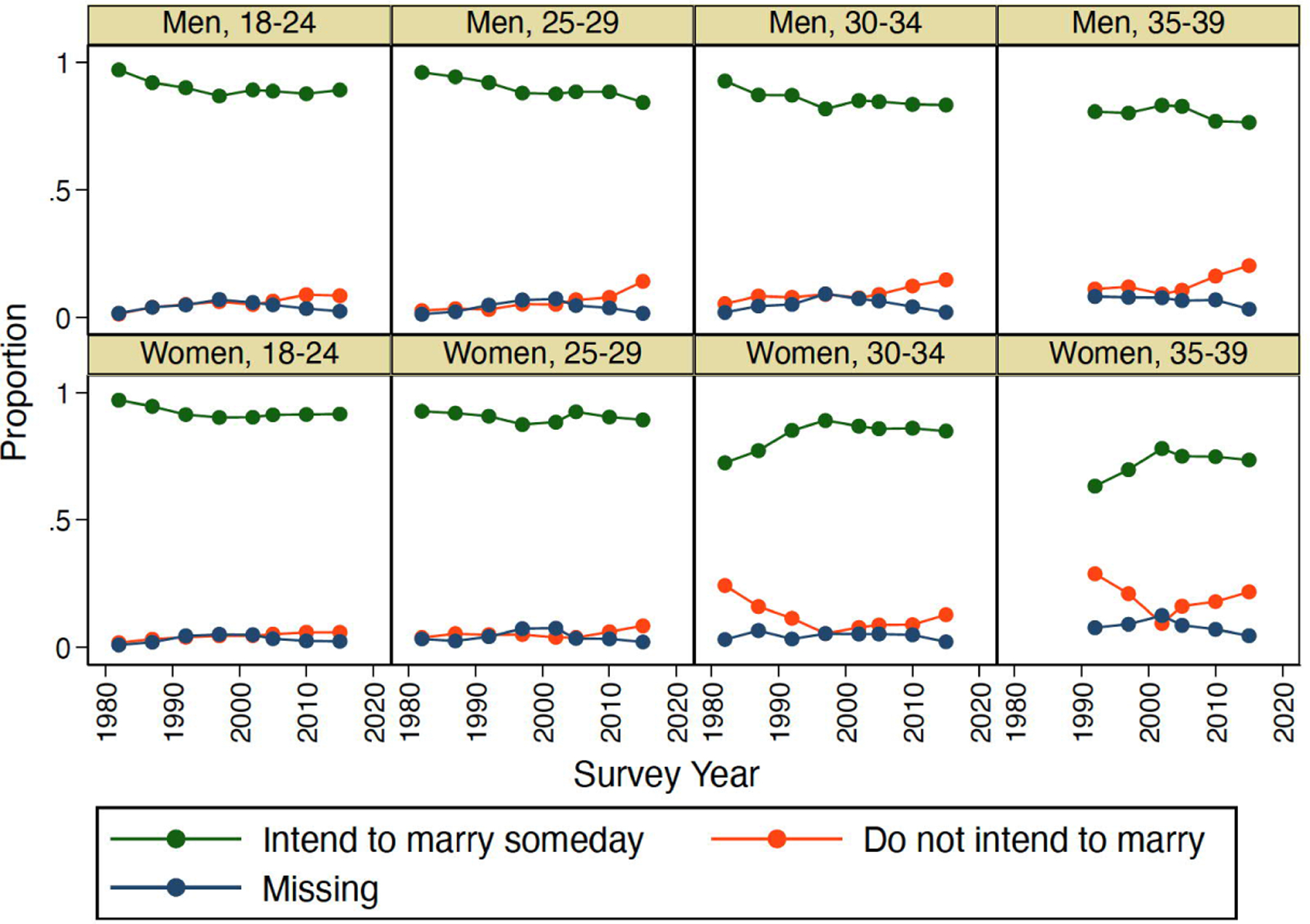
Trends in marriage intentions by sex and age, 1982–2015

Figure 1 shows trends in marriage intentions of Japanese who have never been married between 1982 and 2015 by sex and age based on the yes-no question in the JNFS. Overall, the proportion not intending to marry (consistent with rejection of marriage) is low but has increased somewhat over time. Looking first at men, we see that this trend is particularly pronounced at older ages, with the proportion of 30- to 34-year-old men not intending to marry increasing from .05 in 1982 to .15 in 2015. The pattern for unmarried women is different. On one hand, the proportion of younger women (ages 18 to 24) that does not intend to marry has remained small, increasing from .02 in 1982 to .06 in 2015. On the other hand, the proportion of older women (ages 30 to 39) intending to marry increased during the 1980s and the 1990s (accompanied by a corresponding decrease in the proportion intending to never marry). These trends presumably reflect the increasing age at marriage and associated change in the composition of older women who have never married with respect to marriage intentions.

Interestingly, however, the proportion of older unmarried women intending to marry has been stable since the late 1990s, while the proportion that does not intend to marry increased (from .09 in 2002 to .22 in 2015 among 35- to 39-year-old women who have never been married). This trend primarily reflects a decline in the proportion of women with missing values on the marriage intentions question, a pattern that hints at the possibility of weakening normative expectations or pressure to marry being reflected in an increased willingness to express intentions not to marry in the context of a social survey. Overall, the trends described in Figure 1 suggest that, although the large majority of young men and women in Japan intend to marry, there is some evidence of an increase in the relatively small proportions that appear to be rejecting marriage.

**Figure 2:**
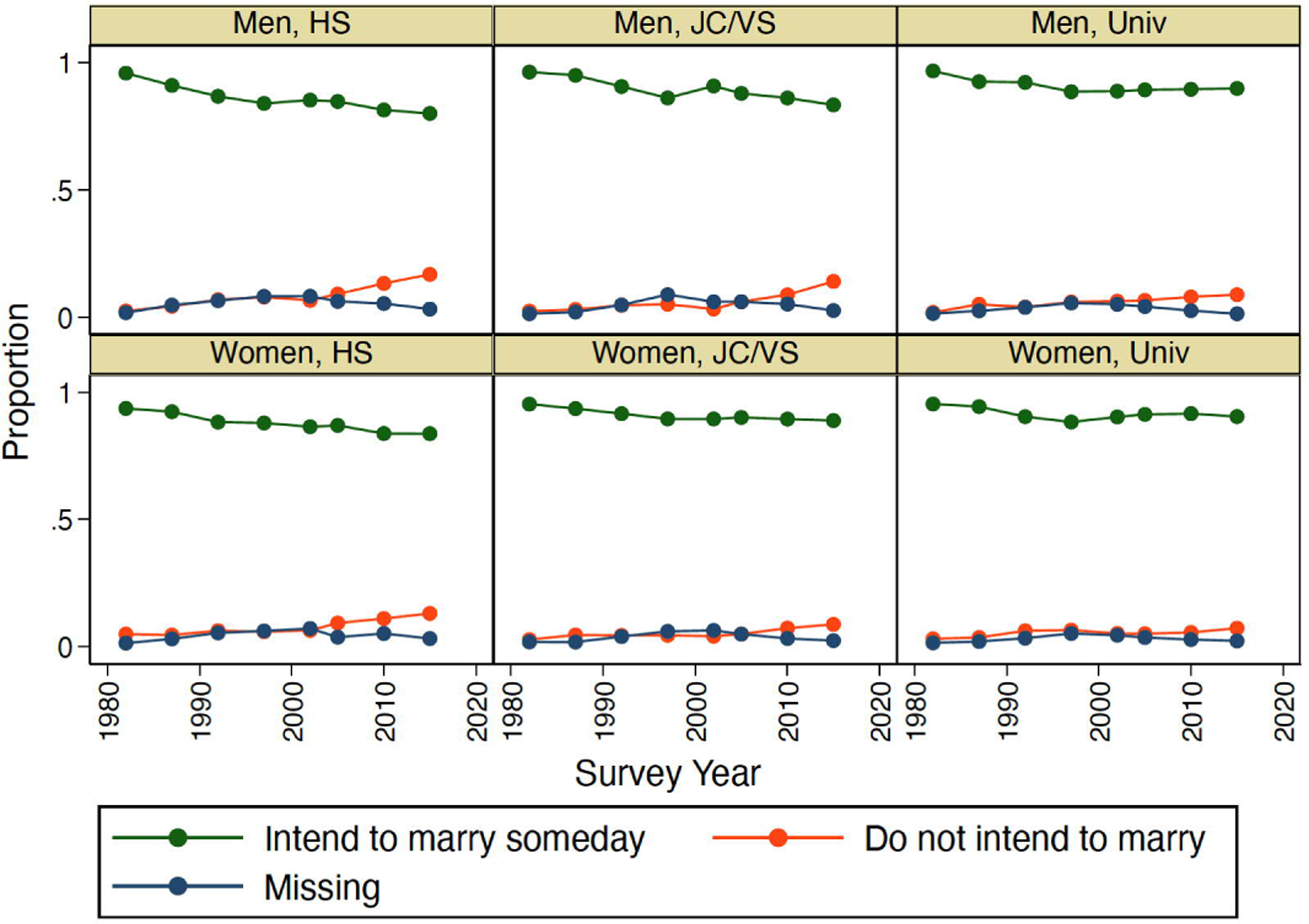
Trends in marriage intentions by sex and educational attainment, 1982–2015 *Note*: HS=High school or less, JC/VS=Junior college/Vocational school, Univ=University or more.

**Figure 3:**
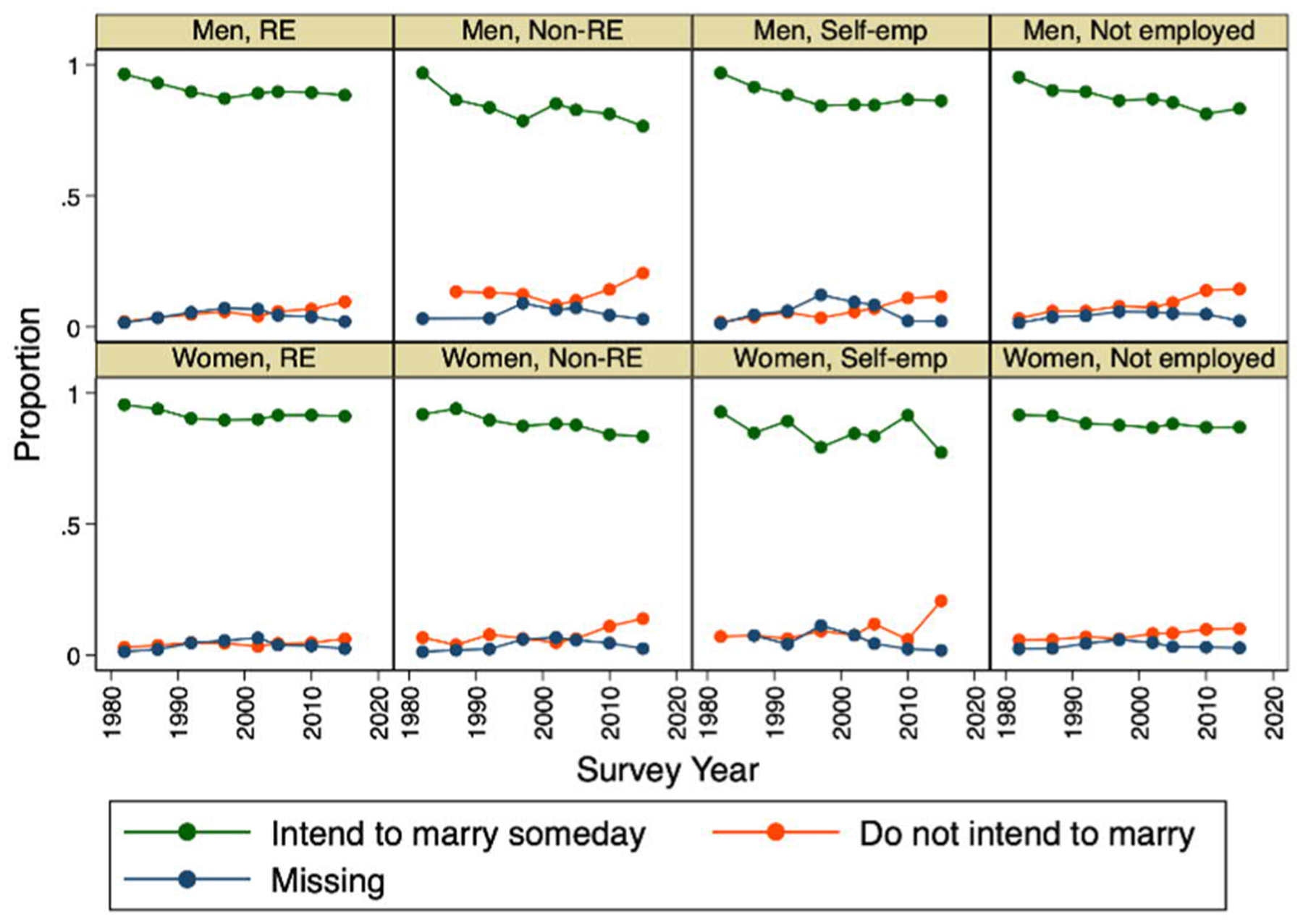
Trends in marriage intentions by sex and employment status, 1982–2015 *Note*: RE=Regular/full-time employment, Non-RE=Nonstandard/part-time employment, Self-emp=Self-employment.

Figures 2 and 3 describe trends in marriage intentions by educational attainment and employment status, respectively. In contrast to the relatively stable marriage intentions of men with university education and regular employment, those with less education and in nonstandard work are increasingly likely to express intentions not to marry. The patterns are generally similar for women, with little change in the high prevalence of intentions to marry among the highly educated and those in either regular employment or not working and some decline among those with low education and in nonstandard employment. None of these data are consistent with expectations of the rejection of marriage scenarios in which intentions to remain unmarried are concentrated among women with the highest opportunity costs of marriage and more attractive alternatives to marriage.

### 4.2 Japanese Life Course Panel Survey

Table 2 presents the distribution of marriage desires of unmarried men and women age 20 to 40 in the first wave (2007) of the JLPS by age, education, and employment status. Again, we see that desires to remain unmarried are uncommon – less than 3% of men and women reported that they “don’t want to marry.” Three-fourths of both men and women reported that they want to marry, with about half of this group saying “definitely” and the other half saying “if possible.” Sizable proportions reported either ambivalent desires or low interest in marriage (23% of men and 18% of women). Actively positive desires to marry (i.e., “definitely want to marry”) are negatively associated with age for both men and women, a pattern that is consistent with selective survival (in the state of never having married) of those with weaker marriage desires and perhaps with a lowering of expectations (desires) for marriage as respondents age. Similar explanations may account for the relatively high proportions of older singles with passively positive desires (“want to marry if possible”), ambivalent desires (“don’t care if marry or not”), and negative desires (“don’t want to marry”). It is perhaps not surprising that younger respondents are much more likely to indicate that they are “not thinking about marriage” given the erosion of norms regarding marriageable age and the increasingly older ages at which men and women marry.

Marriage desires are clearly related to educational attainment, especially for men. Highly educated men are much more likely to express clear desires to marry than their less-educated counterparts, who are more likely to respond that they “would like to marry if possible” or are “not thinking about marriage.” Educational differences in desires are similar, but not as pronounced, for women who have never married. In contrast, differences in marriage desires by employment status are small for men but pronounced for women. Women who are not employed are most likely to express actively positive desires to marry (perhaps reflecting labor force exit in anticipation of marriage) and women in nonstandard/part-time employment are the most likely to express passively positive desires or low interest in marriage (i.e., “would like to marry if possible” and “not thinking about marriage”).

Table 3 presents a simple descriptive picture of the stability of marriage desires, tabulating desires at waves *t* and *t*+1 (wave *t*+1 categories include ‘married’ as a destination). It is not surprising that the largest cells are on the main diagonal (i.e., stable desires), but the fact that only about half of respondents reported the same marriage desires in consecutive years points to the fluidity of desires. This is especially true for ambivalent desires, low interest, and negative marriage desires – stability was highest for “definitely want to marry” and “want to marry if possible” and those who were in one of these categories at wave *t* tended to move to the other category in wave *t*+1 (this was true for 19% of those who said “definitely want to marry” and 20% of those who responded “want to marry if possible”). It is also not surprising, given the ordinal nature of these desire categories, that cross-wave changes tended to involve movement to an adjacent category and that transition to marriage was highest among those with stronger desires to marry (13% of those who said that they “definitely want to marry” were married at the next wave of the survey). It is interesting that, among respondents in the intermediate desire categories, cross-wave movement toward more positive desires was more common than the reverse.

**Figure 4:**
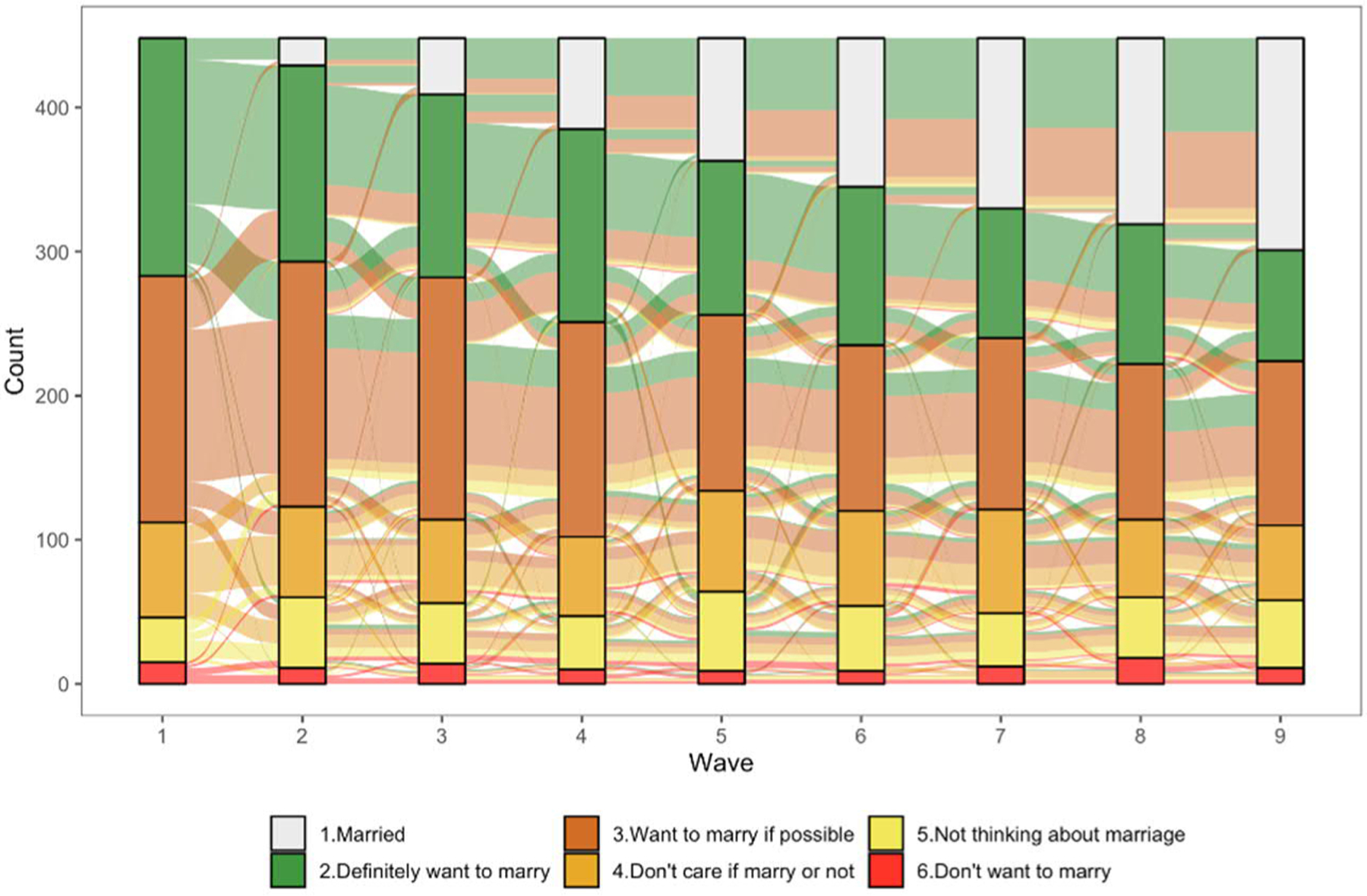
Trajectories of men’s marriage desires, waves 1–9

**Figure 5:**
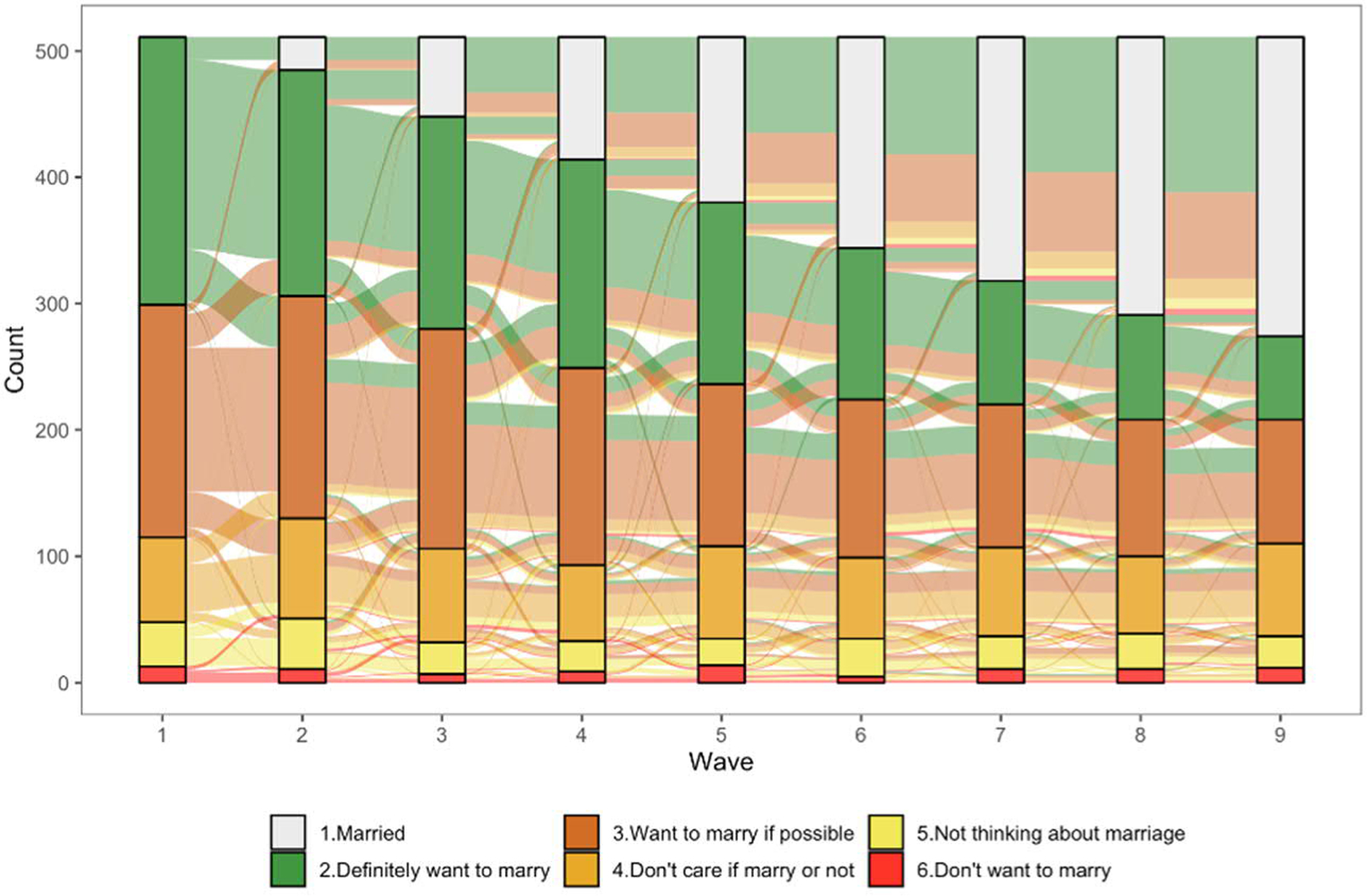
Trajectories of women’s marriage desires, waves 1–9

Figures 4 and 5 present flows of marriage desires for men and women, respectively, across all nine waves of available JLPS data using alluvial plots. These figures describe the responses of those who were never married at wave 1 and responded in all subsequent waves (2 through 9) of the survey (448 male and 511 female respondents). Categories are ordered from strongest to weakest marriage desires (with married at the top), and each category is represented by a different color, with flows colored to describe trajectories by marriage desires stated at wave 1 (e.g., the green colored flows represent the pathways of those who responded “definitely want to marry” at wave 1).^9^

As already shown in Tables 2 and 3, evidence of a rejection of marriage is minimal, with very small proportions of men and women consistently responding that they do not want to marry (indicated in red in Figures 4 and 5). The fact that a nontrivial percentage of the small number who said that they “don’t want to marry” at wave 1 were actually married by wave 9 (9% for men and 21% for women) is also not consistent with a scenario characterized by rejection or opting out of marriage. Among those with actively positive desires (“definitely want to marry”) at wave 1, 46% of men and 61% of women had married by wave 9. Among those who said “definitely want to marry” at wave 1 and remain unmarried, nearly all consistently reported that they definitely want to marry or want to marry if possible. While we obviously cannot determine if these unmarried respondents will ultimately marry or not, they are candidates for the ‘failure to realize marriage desires’ pathway. This is a sizable group, with 22% of men and 17% of women never married at wave 9 either consistently in one of these groups or moving from one to the other.

Another sizable group is those who consistently report a desire to marry if possible or move from this group to ambivalent desires or low interest. While a nontrivial proportion of those who expressed passively positive desires, ambivalent desires, or low interest at wave 1 were married by wave 9 (26% of men and 35% of women), those remaining unmarried are candidates for the pathway we describe as unplanned drifting into singlehood (41% of men and 34% of women who remained never married at wave 9).

To simplify the complex trajectories depicted in Figures 4 and 5 and to quantify the prevalence of the three patterns outlined in Table 2, by SES, we allocated respondents to one of five trajectory groups at each wave of the JLPS based on their marriage desires at the previous wave. As described in Table A–1 in the Appendix, the allocation procedure is such that ‘rejection of marriage’ refers to those who remain unmarried and report “don’t want to marry” (regardless of stated desires at the previous wave), ‘failure to realize marriage desires’ refers to those who remain unmarried despite definitely wanting to marry (or moving between “definitely want to marry” and “want to marry if possible”), and unplanned drifting includes those who are consistently in, or move among, one of the intermediate categories of “marry if possible,” “don’t care,” and “not thinking about marriage.”^10^ Based on this theoretically informed, but analytically unsophisticated, classification scheme, we calculate that the proportion of men in each pathway at wave 9 is 33% married, 3% rejection, 22% failure to realize desires, and 41% unplanned drifting. The corresponding figures for women are 46%, 2%, 17%, and 34%.^11^ As shown in Table A–2, the distributions of pathways are remarkably similar for men and women and differ somewhat depending on educational attainment and wave of the survey. Among those who remain unmarried, the prevalence of ‘drifting’ is higher among both men and women who did not complete a four-year university education and the prevalence of ‘drifting’ among singles increases with age (time).^12^

Tables 4 and 5 present the results of the discrete-time complementary log-log models for the transition to marriage focusing on differences by educational attainment and employment status, respectively. In these models, we are interested in evaluating (a) the degree to which differences in marriage desires account for observed differences by socioeconomic status in marriage rates and (b) whether the relationship between marriage desires and the transition to marriage differs by socioeconomic status. Results of these models, estimated separately by sex, provide an empirical basis for evaluating the theoretically informed pathway-specific predictions in column 4 of Table 1.

The baseline hazard is modeled as a quadratic function of age, and the first model includes living arrangements (living with parents or not), region of residence (large metropolitan area, other city, and other), and either educational attainment (Table 4) or employment status (Table 5). All variables are measured at wave *t*−1 with the exception of educational attainment which is fixed at its wave 1 value. In Model 2, we include marriage desires measured at wave *t*−1 and assess the degree to which desires matter by comparing the coefficients for socioeconomic status in Models 1 and 2.^13^ In Model 3, we include interactions between marriage desires and the measures of socioeconomic status to evaluate the posited differences in relationships between marriage desires and transitions.

Looking first at Table 4, we see that education is unrelated to women’s risk of marriage in this sample, and university-educated men’s risk of marriage (= 0.33) is higher than those who completed high school or less, findings that are similar to those from Fukuda, Raymo, and Yoda’s (2020) analyses of data from the JNFS but should be interpreted cautiously as the 95% confidence interval includes zero. Model 2 shows that, after controlling for stronger marriage desires among the highly educated, women with at least a two-year college degree had a lower risk of marriage than those who only completed high school (or less) (95% CI are −0.69 to −0.08 for junior college/vocational school and −0.70 to −0.11 for university or more) and men’s educational attainment was no longer associated with marriage.^14^ This pattern is most consistent with the unplanned drifting scenario in which women and men with lower levels of education are less likely to marry as a result of having weaker desires to marry than their highly educated counterparts. However, Model 3 provided no evidence that educational attainment is associated with the likelihood of realizing marriage desires; none of the interaction terms were different from zero.

The pattern of results was very similar in Table 5 for women but not for men. The positive association between regular employment (relative to nonstandard employment) and marriage for women in Model 1 was no longer different from zero in Model 2, reflecting the stronger marriage desires of those in regular employment. For men, the strong positive association between regular employment and marriage in Model 1 was attenuated somewhat after controlling for the stronger marriage desires among these men, but this difference remained different from zero [95% CI of 0.27 to 1.19]. The interactions included in Model 3 provided almost no evidence of socioeconomic differences in the relationship between marriage desires and marriage. One exception showed women in regular employment who reported ambivalent attitudes about marriage were less likely to marry (= −0.89). While this result is consistent with predictions derived from gender equity theory and its emphasis on the either-or choice between work and family for women, especially for women with greater opportunities in the work sphere, we need to interpret it cautiously as the 95% confidence interval includes zero.

## 5. Discussion

Theoretical frameworks for understanding later and less marriage in Japan (and elsewhere) regularly reference marriage attitudes, desires, and intentions, but empirical studies typically have not considered marriage intentions or desires, their (in)stability across young adulthood, or their relationships with marriage outcomes. Our goal in this paper was to address these limitations in an effort to generate new insights into the social and economic processes underlying the trend toward later and less marriage in Japan. We focused in particular on three possible patterns of marriage desires and outcomes, or “pathways” to later and less marriage: (1) rejection of marriage, (2) failure to realize intentions to marry, and (3) unplanned ‘drifting’ into singlehood.

While previous research and popular media accounts have regularly emphasized women’s growing economic independence and how it allows them to opt out of gender-inegalitarian marriages, our results clearly demonstrate that there is little reason to believe that later and less marriage in Japan primarily reflects active rejection of marriage on the part of either women or men. This does not mean that rejection of marriage is irrelevant, but it does mean that the prevalence of this pathway to lifelong singlehood is low (especially when respondents are given the option to choose intermediate intentions such as “not thinking about marriage” or “don’t care if I marry or not”). It is also true that many respondents who say that they do not intend to marry believe that they may change their mind later (IPSS 2017b) and Figures 4 and 5 demonstrate that a nontrivial proportion of young adults (especially women) who initially reported not wanting to marry eventually do marry. To some degree, this pattern may simply reflect the process of aging among younger men and women who expressed desires to never marry at wave 1 (the same pattern holds for younger men and women who indicated that they were “not thinking about marriage” at wave 1). At the same time, we might also interpret this pattern of results as a reflection of normative pressure or expectations to marry, especially in light of the still limited range of romantic relationship alternatives to marriage in Japan. This pattern applies to only a small segment of the population, but it also suggests the relevance of social and economic forces that may minimize the likelihood of an active ‘retreat from marriage.’

Our scheme for classifying respondents into three different pathways that might lead to lifelong singlehood shows that unplanned drifting and failure to realize desires to marry are both quite common. Among JLPS respondents who remain unmarried at wave 9, roughly two-thirds can be classified as ‘drifting,’ about 30% as ‘failing to realize marriage desires,’ and no more than 5% as ‘rejecting marriage.’ As described in the background section, the high prevalence of ‘failing to realize marriage desires’ is consistent with theoretical emphases on both economic uncertainty and difficulties meeting potential partners. The even higher prevalence of ‘drifting’ is consistent with theoretical emphases on extended spouse search and ambivalence about marriage in settings where work–family balance is particularly difficult. It is also consistent with context-specific emphases on the comforts of home and the comforts of single life more generally.

Our analyses suffer from many limitations. First and foremost, the fact that many of the JLPS respondents are still relatively young at wave 9 of the survey (the youngest are 29 years old) means that we cannot directly examine lifelong singlehood. Rather, this is a study of the pathways that may result in lifelong singlehood for some proportion of the respondents who had yet to marry when we last observed them. Second, small sample sizes limit our ability to estimate relationships of interest with precision using the JLPS data. This is particularly problematic for estimation of coefficients for the rare transitions to marriage among those who express low interest in marriage or a desire to remain unmarried. Third, the simple measures of marriage intentions and desires available in the JNFS and JLPS, respectively, tell us nothing about the context in which respondents are answering and likely obscure a good deal of important variation in the nature of past and ongoing romantic relationships and the presence or absence of specific plans for marriage. We are unaware of any source of existing data that would allow us to fully address this limitation (but see Motegi and Ishida 2019 for an examination of marriage desires and dating relationships using the JLPS data). Fourth, we do not actually observe the various factors central to theoretical explanations of later and less marriage (e.g., economic insecurity at young ages, the convenience and comfort of single life, and the difficulty of meeting potential partners).

Despite these limitations, we see value in this effort to comprehensively describe marriage intentions/desires and their association with marriage outcomes in a setting where low rates of marriage and projected growth in lifelong singlehood are major policy issues. Of particular importance, from both a theoretical and a substantive perspective, is our provision of compelling empirical evidence that patterns of ‘drifting’ into singlehood and failing to realize marriage desires are both important. These results suggest the possibility of a ‘perfect storm’ in which a combination of social and economic factors result in both failure to realize marriage desires and unplanned drifting into singlehood (and to a much lesser degree, rejection of marriage). Given the fluidity of marriage desires described in Table 3, the results of the transition rate models in Tables 4 and 5 highlight the importance of further efforts to understand the life circumstances and experiences associated with the contemporaneous expression of marriage intentions and desires (e.g., economic well-being, employment satisfaction, personal goals and activities, and presence of a partner).

## Supplementary Material

Data and Read Me Files

## Figures and Tables

**Table 1:** Hypothesized patterns of marriage intentions/desires for the three pathways to later and less marriage

Pathway to later and less marriage	(1) Marriage intentions/desires	(2) SES differences	(3) Trajectories	(4) Outcomes
Rejection of marriage	Intend/desire to remain unmarried	Concentrated among high-SES women and highly educated men	Stable intentions/desires to remain unmarried	Intentions/desires to remain unmarried account for lower marriage rates among high-SES women and highly educated men
Failure to realize intentions/desires to marry	Intend/desire to marry	Higher among low-SES women and men	Stable intentions/desires to marry	Stronger intentions/desires to marry account for higher rates of marriage among low-SES women. Lower likelihood of realizing intentions/desires among low-SES men and women
Unplanned drifting into singlehood	Passive or ambivalent marriage intentions/desires	Mixed: (a) higher among high-SES in search theory and gender equity theories, (b) higher among low-SES in emphases on passivity	Stable passive or ambivalent intentions/desires (or inconsistent intentions/desires)	Could account for lower marriage rates among both high-SES and low-SES men and women. Lower likelihood of realizing passive intentions/desires among low-SES men and women

**Table 2:** Descriptive statistics by sex at wave 1 JLPS (as percent)

	Definitely want to marry	Want to marry if possible	Don’t care if marry or not	Not thinking about marriage	Don’t want to marry
*Men (n* = *1,247)*					
*Total*	37.3	37.5	13.4	9.1	2.6
*Age*					
20–24	41.4	36.6	10.4	9.3	2.3
25–29	40.2	33.7	12.4	11.5	2.2
30–34	33.9	41.8	14.9	7.3	2.2
35–40	27.8	39.2	19.9	8.0	5.1
*Educational attainment*					
High school or less	30.9	40.2	14.5	12.1	2.3
Two-year college	30.3	39.0	16.1	11.0	3.5
University or more	43.4	35.5	11.7	6.8	2.5
*Employment status*					
Regular/full-time	39.4	37.3	14.7	6.7	1.8
Nonstandard/part-time	32.7	38.6	9.9	15.3	3.5
Self-employed	33.8	39.4	14.1	8.5	4.2
Not employed	36.6	37.0	12.7	10.2	3.4
*Women (n = 1,091)*					
*Total*	44.2	34.9	12.7	5.7	2.5
*Age*					
20–24	53.1	31.6	8.5	4.8	2.1
25–29	45.1	37.5	10.5	5.7	1.3
30–34	41.4	31.8	19.2	4.0	3.5
35–40	18.4	44.0	22.0	10.6	5.0
*Educational attainment*					
High school or less	36.5	35.2	14.2	11.0	3.2
Two-year college	43.3	36.4	11.6	6.2	2.5
University or more	49.1	33.3	13.2	2.4	2.1
*Employment status*					
Regular/full-time	45.4	34.2	12.7	4.8	2.9
Nonstandard/part-time	37.7	39.7	13.0	7.3	2.3
Self-employed	40.0	28.0	24.0	4.0	4.0
Not employed	50.0	31.2	11.2	5.8	1.7

**Table 3:** Marriage desires at wave *t* and wave *t*+1 (percent)

Wave *t* /Wave *t*+1	Definitely want to marry	Want to marry if possible	Don’t care if marry or not	Not thinking about marriage	Don’t want to marry	Married	Total	N
Definitely want to marry	65.9	18.6	1.7	1.2	0.2	12.5	100	3,564
Want to marry if possible	19.8	61.1	10.0	4.4	0.3	4.4	100	3,578
Don’t care if marry or not	3.2	21.6	54.5	14.9	3.6	2.1	100	1,539
Not thinking about marriage	3.9	17.0	23.2	48.3	7.2	0.6	100	907
Don’t want to marry	2.2	6.2	18.3	22.3	51.1	0.0	100	274
Total	27.7	29.0	12.8	7.8	2.3	20.5	100	9,862

**Table 4:** Estimated coefficients from complementary log-log models for the transition to first marriage (SES = Educational attainment)

Variable	Men			Women		
	Model 1	Model 2	Model 3	Model 1	Model 2	Model 3
Age	0.38 [0.12,0.64]	0.39 [0.13,0.66]	0.4 [0.13,0.66]	1.18 [0.89,1.47]	1.16 [0.87,1.45]	1.16 [0.87,1.45]
Age squared	−0.01 [−0.01,−0.00]	−0.01 [−0.01,−0.00]	−0.01 [−0.01,−0.00]	−0.02 [−0.02,−0.01]	−0.02 [−0.02,−0.01]	−0.02 [−0.02,−0.01]
*Living arrangements* (*Reference: Living independently*)						
Coresidence with parents	−0.83 [−1.10,−0.56]	−0.81 [−1.08,−0.53]	−0.81 [−1.08,−0.54]	−0.42 [−0.66,−0.18]	−0.39 [−0.63,−0.15]	−0.39 [−0.63,−0.15]
*Region of residence* (*Reference: Major metropolitan area*)						
Large city	0.15 [−0.19,0.49]	0.12 [−0.22,0.46]	0.12 [−0.22,0.46]	0.13 [−0.15,0.41]	0.11 [−0.17,0.39]	0.1 [−0.18,0.39]
Other	0.03 [−0.28,0.34]	0.06 [−0.25,0.37]	0.06 [−0.25,0.38]	0.07 [−0.19,0.33]	0.06 [−0.19,0.32]	0.06 [−0.20,0.31]
*Educational attainment* (*Reference: High school or less*)						
Junior college/Vocational school	0.26 [−0.17,0.69]	0.29 [−0.14,0.72]	0.35 [−0.17,0.87]	−0.24 [−0.54,0.06]	−0.39 [−0.69,−0.08]	−0.32 [−0.70,0.05]
University or more	0.33 [−0.03,0.70]	0.24 [−0.12,0.61]	0.35 [−0.09,0.79]	−0.17 [−0.46,0.13]	−0.4 [−0.70,−0.11]	−0.41 [−0.78,−0.05]
*Marriage desires* (*Reference: Definitely want to marry*)						
Want to marry if possible		−1.26 [−1.57,−0.94]	−0.96 [−1.62,−0.29]		−0.98 [−1.24,−0.73]	−0.91 [−1.44,−0.38]
Don’t care if I marry or not		−2.22 [−2.93,−1.51]	−2.47 [−3.63,−1.31]		−1.54 [−2.02,−1.07]	−1.56 [−2.49,−0.62]
Not thinking about marriage/Don’t want to marry					−3.31 [−4.70,−1.91]	−3.74 [−5.72,−1.77]
*Marriage desires* × *Educational attainment*						
Jr. college/Voc. × Want to marry if possible			−0.19 [−1.13,0.75]			−0.28 [−0.96,0.40]
Jr. college/Voc. × Don’t care if marry or not			0 [0.00,0.00]			0.16 [−1.03,1.34]
Jr. college/Voc. × Not thinking/don’t want to marry			–			–
University × Want to marry if possible			−0.46 [−1.25,0.33]			0.07 [−0.60,0.73]
University × Don’t care if marry or not			0.46 [−1.01,1.93]			−0.14 [−1.38,1.11]
University × Not thinking/don’t want to marry						1.21 [−1.57,4.00]
Constant	−8.45 [−12.7,−4.2]	−8.26 [−12.5,−4.0]	−8.42 [−12.7,−4.1]	−19.86 [−24.3,−15.4]	−19.33 [−23.8,−14.8]	−19.31 [−23.8,−14.8]
Observations	4,128	4,128	4,128	4,579	4,579	4,579
Log-likelihood	−833.32	−777.11	−776.12	−1122.24	−1051.74	−1050.46

*Note*: Bracketed values show the 95% confidence interval.

**Table 5:** Estimated coefficients from complementary log-log models for the transition to first marriage (SES=Employment status)

Variable	Men			Women		
	Model 1	Model 2	Model 3	Model 1	Model 2	Model 3
Age	0.12 [−0.15,0.39]	0.14 [−0.13,0.42]	0.14 [−0.13,0.42]	1.07 [0.77,1.36]	1.04 [0.75,1.34]	1.03 [0.74,1.33]
Age squared	0 [−0.01,0.00]	0 [−0.01,0.00]	0 [−0.01,0.00]	−0.02 [−0.02,−0.01]	−0.02 [−0.02,−0.01]	−0.02 [−0.02,−0.01]
*Living arrangements* (*Reference: Living independently*)						
Coresidence with parents	−0.81 [−1.08,−0.54]	−0.78 [−1.05,−0.51]	−0.78 [−1.06,−0.51]	−0.4 [−0.64,−0.16]	−0.35 [−0.59,−0.11]	−0.35 [−0.59,−0.11]
*Region of residence* (*Reference: Major metropolitan area*)						
Large city	0.13 [−0.22,0.47]	0.09 [−0.25,0.43]	0.09 [−0.25,0.44]	0.14 [−0.15,0.42]	0.13 [−0.15,0.41]	0.13 [−0.15,0.41]
Other	−0.09 [−0.40,0.22]	−0.06 [−0.37,0.25]	−0.06 [−0.37,0.24]	0.08 [−0.18,0.33]	0.09 [−0.17,0.34]	0.09 [−0.17,0.34]
*Employment status* (*Reference: Nonstandard/part-time*)						
Regular/full-time	0.83 [0.37,1.29]	0.73 [0.27,1.19]	0.7 [0.16,1.24]	0.22 [−0.03,0.47]	0.09 [−0.16,0.34]	0.13 [−0.18,0.44]
Self-employed	0.66 [−0.01,1.33]	0.73 [0.06,1.40]	0.67 [−0.13,1.48]	−0.29 [−1.12,0.54]	−0.16 [−0.99,0.67]	−0.2 [−1.21,0.81]
Not employed	−1.57 [−2.66,−0.48]	−1.63 [−2.72,−0.54]	−2.11 [−3.60,−0.62]	−0.76 [−1.32,−0.21]	−0.72 [−1.27,−0.16]	−0.72 [−1.40,−0.04]
*Marriage desires* (*Reference: Definitely want to marry*)						
Want to marry if possible		−1.25 [−1.56,−0.93]	−13 [−2.31,−0.29]		−0.95 [−1.21,−0.70]	−0.97 [−1.46,−0.47]
Don’t care if 1 marry or not		−2.19 [−2.91,−1.48]	−2.87 [−4.87,−0.87]		−1.47 [−1.94,−0.99]	−1.14 [−1.81,−0.46]
Not thinking about marriage/Don’t want to marry					−3.16 [−4.56,−1.77]	−3.16 [−4.55,−1.76]
*Marriage desires* × *Employment status*						
Regular × Want to marry if possible			0 [−1.07,1.08]			0.04 [−0.54,0.63]
Regular × Don’t care if marry or not			0.83 [−1.31,2.97]			−0.89 [−1.95,0.16]
Self-employed × Want to marry if possible			0.22 [−1.25,1.70]			0.08 [−1.69,1.85]
Not employed × Want to marry if possible			1.39 [−0.82,3.59]			−0.52 [−2.12,1.08]
Not employed × Don’t care if marry or not						0.62 [−0.84,2.07]
Constant	−4.3 [−8.7,0.1]	−4.22 [−8.7,0.3]	−4.17 [−8.6,0.3]	−18.31 [−22.9,−13.8]	−17.82 [−22.4,−13.2]	−17.71 [−22.3,−13.1]
Observations	4,128	4,128	4,128	4,579	4,579	4,579
log-likelihood	−807.43	−752.61	−751.28	−1113.77	−1049.84	−1046.89

*Note*: Bracketed values show the 95% confidence interval. The interaction between ‘self-employed’ and ‘don’t care if marry or not’ was not included because it predicts the outcome perfectly.

## Footnotes

4 As described below, we consider three specific pathways – rejection of marriage, failure to realize desires to marry, and unplanned drifting into singlehood. The first encompasses those who do not want to marry and follow through on those desires. The second is those who, despite a clear desire to marry, remain unmarried. The third is those who express ambivalence or low interest in marriage while remaining unmarried.

5 Desires and intentions are not the same. Desires are thought to reflect underlying attitudes and predispositions, whereas intentions represent planned behavior based on existing desires and perceived constraints on realizing those desires (e.g., Miller 2011; Thomson 1997). In this paper, we examine measures of both marriage intentions and marriage desires. We discuss both in the background and distinguish between the two in discussions specific to the data we analyze. Throughout, we assume that relationships of interest are similar for marriage intentions and desires.

6 Conventional articulations of this model emphasize economic opportunity costs (i.e., lost wages), but the same argument would apply to a broader definition of opportunity costs, perhaps including freedom, leisure, and the meaning and rewards derived from work.

7 While it is true that writing on women’s economic independence and marriage says little about men’s views of marriage, it is important to recognize that marriages characterized by gender-based specialization may also be increasingly unattractive to men unwilling or unable to specialize in the role of primary breadwinner. Recent attitudinal data indicate that preferences for a “traditional” breadwinner-homemaker division of labor is declining in Japan for both women and men (IPSS 2017b).

8 We excluded respondents with missing information on educational attainment or employment status (4.7% of the total sample of respondents who have never been married).

9 Here, married is an absorbing state and thus encompasses all respondents who ever married, including those who subsequently divorced or were widowed.

10 Because we do not observe respondents into midlife, it is more appropriate to call the second group ‘not yet realized marriage desires’ rather than ‘failure to realize marriage desires.’

11 These percentages do not sum to 100 because there is a small number whose pattern of intentions could not be easily summarized using these four groups (see Table A–1 in the Appendix).

12 The time-varying nature of employment status limits the value of constructing similar tabulations by that dimension of socioeconomic status.

13 Our decision to use only a single measure of marriage intentions (at wave *t*−1) was motivated by evidence from auxiliary models indicating that, net of temporally proximate desires, earlier measures of marriage desires were unrelated to the risk of marriage. We do not focus on this finding but consider it important for understanding the nature and implications of marriage desire trajectories.

14 We combined “not thinking about marriage” and “don’t want to marry” into a single category given the very small number of marriages for respondents who reported either of these intentions. The coefficient for this category is constrained to equal zero in models for men because no respondent in the category at wave *t*−1 married by wave *t*.

## Appendix

**Table A-1:** Classification of respondents into five marriage desire trajectory groups

Assignment criteria		
Trajectory group	Desires at wave *t*	Desires at wave *t*-1
1) Married	Married	Any desire
2) Failure to realize marriage desires	“Definitely want to marry”	“Definitely want to marry” or “Want to marry if possible”
	“Want to marry if possible”	“Definitely want to marry”
3) Unplanned drifting	“Want to marry if possible”	“Want to marry if possible,” “Don’t care if I marry,” or “Not thinking about marriage”
	“Don’t care if I marry”	“Want to marry if possible,” “Don’t care if I marry,” or “Not thinking about marriage”
	“Not thinking about marriage”	“Want to marry if possible,” “Don’t care if I marry,” or “Not thinking about marriage”
4) Rejection of marriage	“Don’t want to marry”	“Want to marry if possible,” “Don’t care if I marry,” or “Not thinking about marriage”
5) Unclassified	Any combination not listed above	

**Table A-2:** Distribution of marriage desire trajectory groups by wave, sex, and educational attainment (column percent)

	Wave							
	2	3	4	5	6	7	8	9
**Men (n = 448)**								
Married	4.2	8.7	14.1	19.0	23.0	26.3	28.8	32.8
Unrealized desires	39.1	33.7	32.8	29.2	26.6	24.6	23.2	22.3
Unplanned drifting	51.6	52.2	49.1	46.2	47.1	45.1	42.6	41.3
Rejection of marriage	2.2	3.1	2.0	2.0	2.0	2.7	4.0	2.5
Unclassified	2.9	2.2	2.0	3.6	1.3	1.3	1.3	1.1
**Women (n = 511)**								
Married	5.1	12.3	19.0	25.6	32.7	37.8	43.1	46.4
Unrealized desires	42.7	37.8	37.4	32.1	28.2	22.5	19.0	17.2
Unplanned drifting	49.1	47.4	39.9	37.2	37.4	36.8	34.8	33.7
Rejection of marriage	2.2	1.4	1.8	2.7	1.0	2.2	2.2	2.4
Unclassified	1.0	1.2	2.0	2.4	0.8	0.8	1.0	0.4
**Men, university or more (n = 251)**								
Married	4.4	10.4	14.7	21.9	27.5	31.5	34.3	38.3
Unrealized desires	43.8	35.9	37.1	32.3	27.9	25.1	22.7	22.7
Unplanned drifting	47.4	49.0	43.4	40.6	41.8	38.7	38.3	35.5
Rejection of marriage	1.6	3.2	2.4	2.4	2.0	2.8	3.6	3.2
Unclassified	2.8	1.6	2.4	2.8	0.8	2.0	12	0.4
**Men, less than university (n = 197)**								
Married	4.1	6.6	13.2	15.2	17.3	19.8	21.8	25.9
Unrealized desires	33.0	31.0	27.4	25.4	24.9	23.9	23.9	21.8
Unplanned drifting	56.9	56.4	56.4	53.3	53.8	53.3	48.2	48.7
Rejection of marriage	3.1	3.1	1.5	1.5	2.0	2.5	4.6	1.5
Unclassified	3.1	3.1	1.5	4.6	2.0	0.5	1.5	2.0
**Women, university or more (n = 201)**								
Married	5.0	12.4	17.9	25.4	32.3	38.3	44.8	50.8
Unrealized desires	48.3	45.3	46.8	38.8	32.3	27.9	20.9	17.4
Unplanned drifting	44.8	41.3	32.3	31.8	32.3	31.8	32.3	29.9
Rejection of marriage	1.0	1.0	1.0	2.0	1.5	1.5	1.5	2.0
Unclassified	1.0	0.0	2.0	2.0	1.5	0.5	0.5	0.0
**Women, less than university (n = 310)**								
Married	5.2	12.3	19.7	25.8	32.9	37.4	41.9	43.6
Unrealized desires	39.0	32.9	31.3	27.7	25.5	19.0	17.7	17.1
Unplanned drifting	51.9	51.3	44.8	40.7	40.7	40.0	36.5	36.1
Rejection of marriage	2.9	1.6	2.3	3.2	0.7	2.6	2.6	2.6
Unclassified	1.0	1.9	1.9	2.6	0.3	1.0	1.3	0.7

*Note*: These figures reflect the marriage desires of men and women who were observed in each of the first nine waves of the JLPS.
