# Supplementary figures and images for: Marriage intentions, desires, and pathways to later and less marriage in Japan
Source: Demogr Res. Author manuscript; Available in PMC 2022 Jan 1. (PMC8153689; doi:10.4054/demres.2021.44.3)

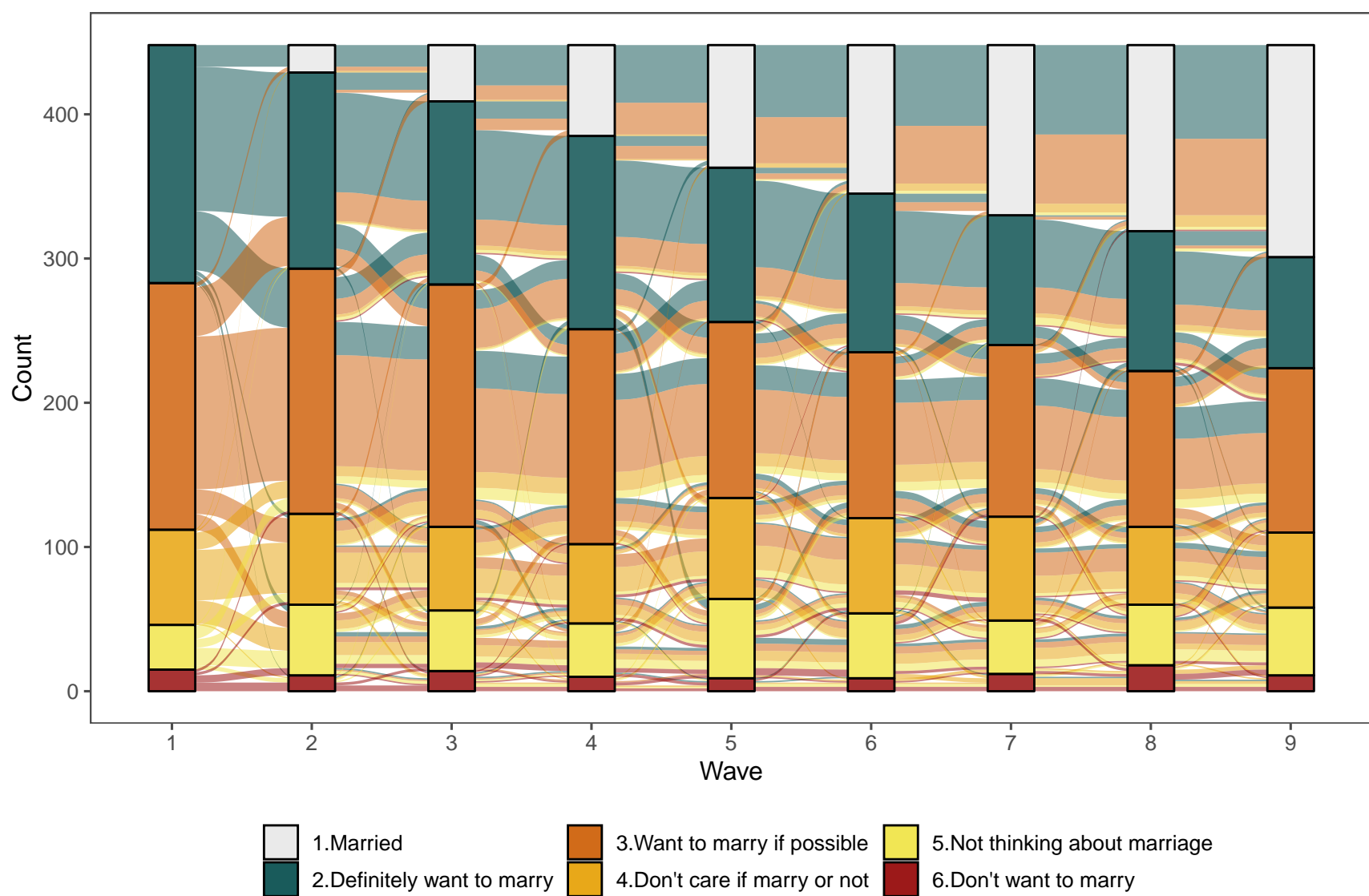

Supplement: Data and Read Me Files [file NIHMS1703303-supplement-Data_and_Read_Me_Files.zip › Results/Figures/Fig_men_w1-9.pdf]

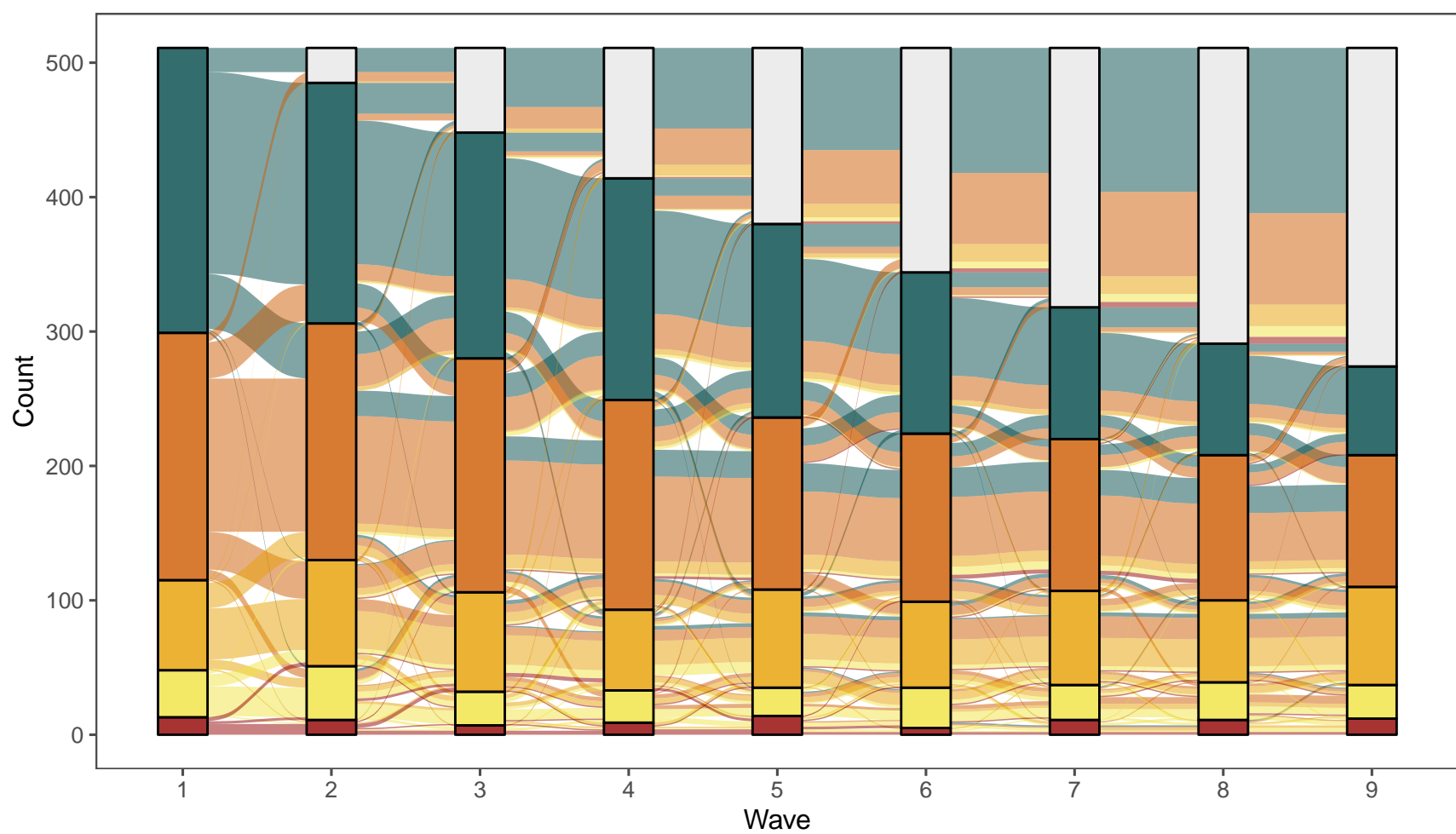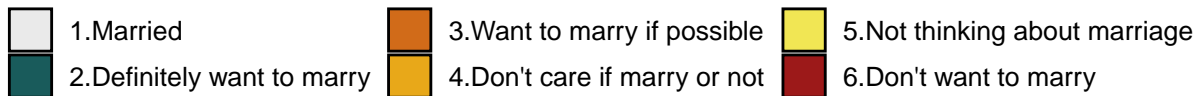

Supplement: Data and Read Me Files [file NIHMS1703303-supplement-Data_and_Read_Me_Files.zip › Results/Figures/Fig_women_w1-9.pdf]
